# U-TXM: a novel biomarker for early detection of diabetic kidney disease

**DOI:** 10.3389/fendo.2025.1682136

**Published:** 2025-11-03

**Authors:** Yujie Jin, Jiahao Xu, Yan Ma, Chunchen Ni, Yan Yao, Shujuan Shang, Mengru Wang, Chunyan Xing, Song Dong, Chaoqun Xiong, Kang Xie, Ruixi Zhang, Wenjun Pei, Shiqiang Liu, Jinhan Cheng, Fan Liu, Siwen Zhang, Dong Li, Lizhuo Wang, Liuming Yu, Jialin Gao

**Affiliations:** ^1^ Department of Endocrinology and Genetic Metabolism, The First Affiliated Hospital of Wannan Medical College (Yijishan Hospital of Wannan Medical College), Wuhu, Anhui, China; ^2^ Institute of Endocrine and Metabolic Diseases, The First Affiliated Hospital of Wannan Medical College, Yijishan Hospital of Wannan Medical College, Wuhu, Anhui, China; ^3^ Anhui Province Key Laboratory of Basic Research and Transformation of Age-related Diseases, Wannan Medical College, Wuhu, Anhui, China; ^4^ Department of Biochemistry and Molecular Biology, Wannan Medical Collage, Wuhu, Anhui, China; ^5^ Suzhou Boyuan Medical Technology Co., Ltd, Suzhou, Jiangsu, China

**Keywords:** diabetic kidney disease, early diagnosis, novel biomarkers, urinary 11-dehydrothromboxane B2, urinary albumin-to-creatinine ratio

## Abstract

**Background and aims:**

Diabetic kidney disease (DKD) is the leading cause of end-stage renal disease. This study aimed to investigate the potential of urinary 11-dehydrothromboxane B2 (U-TXM) as a biomarker for the early detection of DKD.

**Materials and methods:**

A total of 690 patients were enrolled, including 422 with diabetes mellitus (DM) and 268 with DKD. Patients with type 1, type 2, and other specific forms of diabetes were consecutively recruited from the Department of Endocrinology, Yijishan Hospital of Wannan Medical College (April–September 2024). U-TXM levels were measured and their clinical relevance to DKD was evaluated using correlation analysis, logistic regression, and receiver operating characteristic (ROC) curve analysis.

**Results:**

Urinary U-TXM levels were significantly higher in patients with DKD than in those with DM (median: 1158.05 vs. 960.44 pg/mg Cr; *P*<0.001). When stratified by renal function, U-TXM remained elevated in DKD regardless of serum creatinine (Cr) level (>70 or ≤70 μmol/L, both *P*<0.001). Multivariate analysis confirmed the existence of an independent association between DKD and U-TXM (OR=1.778, *P*=0.001), serum Cr (odds ratio [OR]=2.861, *P*<0.001), and systolic blood pressure (SBP, OR=1.032, *P*=0.001). U-TXM correlated positively with the urine albumin-to-Cr ratio (*r*=0.225, *P*<0.001), but only weakly with Cr and blood urea nitrogen. ROC analysis showed limited diagnostic value for U-TXM alone (area under the curve [AUC]=0.625), which improved substantially when combined with serum Cr and SBP (AUC=0.803).

**Conclusion:**

U-TXM shows potential as a biomarker for DKD, particularly in patients at early disease stages. Validation through longitudinal, multicenter, and comparative studies is required to confirm its clinical utility.

## 1 Introduction

Diabetic kidney disease (DKD) is one of the most common microvascular complications of diabetes and remains a leading cause of end-stage renal disease (ESRD) worldwide (1, 2). The pathogenesis of DKD involves hyperglycemia, glomerular hyperfiltration, oxidative stress, and notably, chronic inflammation and immune dysregulation, which are recognized as central drivers of disease progression (3, 4). Hyperglycemia and metabolic imbalance activate several inflammatory signaling cascades—such as NF-κB, JAK/STAT, and TGF-β/Smad—leading to the release of pro-inflammatory cytokines (e.g., TNF-α, IL-1β, IL-6), endothelial activation, leukocyte infiltration, and extracellular matrix accumulation, ultimately contributing to glomerulosclerosis and tubulointerstitial fibrosis (5).

Emerging evidence highlights platelets as active participants in renal inflammation, beyond their traditional role as passive mediators of thrombosis. In diabetic conditions, metabolic stress enhances platelet reactivity and endothelial dysfunction, promoting the release of inflammatory and profibrotic mediators such as thromboxane A_2_ (TXA_2_); platelet factor 4 (PF4); regulated on activation, normal T-cell expressed and secreted; and CD40L (6). These molecules facilitate immune cell recruitment and amplify inflammatory cascades, forming an “immunothrombosis axis” that accelerates renal injury. Thus, platelets represent a critical interface between inflammation, immunity, and fibrosis in the DKD microenvironment (7).

Type 1 diabetes (T1D) typically develops in childhood or adolescence due to autoimmune destruction of pancreatic β-cells, resulting in absolute insulin deficiency and frequent ketoacidosis. In contrast, type 2 diabetes (T2D) is more common in adults and arises from insulin resistance combined with progressive β-cell dysfunction, which is often associated with obesity and metabolic syndrome. Despite these differences, both T1D and T2D can lead to DKD, underscoring the need for reliable biomarkers that capture the shared mechanisms of renal injury.

Current clinical assessment of DKD relies primarily on the urine albumin-to-creatinine (Cr) ratio (UACR) and the estimated glomerular filtration rate (eGFR). However, both indices suffer from considerable limitations. UACR is prone to fluctuation due to hemodynamic changes, infections, or exercise, and may fail to detect structural damage in normoalbuminuric DKD (8, 9). eGFR reflects functional decline but lacks sensitivity for early pathological changes, particularly glomerular inflammation and tubulointerstitial injury (10). Biomarkers like kidney injury molecule-1 and neutrophil gelatinase-associated lipocalin (NGAL) reflect tubular injury but overlook platelet-driven immune activation, a key contributor to DKD progression. Therefore, novel biomarkers are urgently needed to bridge this mechanistic gap and improve early risk stratification.

TXA_2_ is an unstable eicosanoid derived from arachidonic acid, synthesized primarily by activated platelets but also by monocytes and endothelial cells (11). By binding to thromboxane prostanoid receptors, TXA_2_ induces vasoconstriction, glomerular hyperfiltration, leukocyte recruitment, and fibrotic signaling, directly linking thrombosis, inflammation, and fibrosis to renal injury (12–14). Due to its short half-life (~30 seconds), TXA_2_ rapidly hydrolyzes into stable metabolites TXB_2_ and 11-dehydrothromboxane B (U-TXM), the latter serving as a reliable non-invasive marker of *in vivo* TXA_2_ synthesis (15).

Compared with traditional markers, U-TXM offers several advantages: (i) Mechanistic specificity reflecting platelet-immune axis activity; (ii) analytical stability with convenient urinary sampling; (iii) early pathophysiological relevance; and (iv) complementarity with established functional and injury markers. To date, no study has systematically evaluated U-TXM as a mechanism-based biomarker in the context of DKD. In this study, we aim to assess U-TXM as a potential early risk indicator of DKD, highlighting its novelty in bridging platelet-driven inflammation with early renal injury.

## 2 Methods

### 2.1 Research subjects

This study employed a cross-sectional design with prospectively collected clinical and laboratory data. A total of 690 eligible patients were included, comprising 422 with diabetes mellitus (DM) and 268 with DKD. All participants were diagnosed with either type 1, type 2, or other specific forms of diabetes. To ensure representativeness and minimize selection bias, patients were consecutively recruited from the Department of Endocrinology, Yijishan Hospital of Wannan Medical College, between April and September 2024.

The sample size was estimated based on preliminary data comparing urinary U-TXM levels between DM and DKD groups. Assuming a moderate effect size (Cohen’s *d*=0.5), with a two-tailed α of 0.05 and power of 80%, the minimum required sample size was 64 per group. Considering potential dropouts and the need for subgroup analysis, we enrolled a total of 690 participants to ensure adequate statistical power. Sample size calculation was performed using PASS 15.0 software (NCSS, USA) (16).

All participants provided written informed consent prior to enrollment. The study protocol was approved by the institutional ethics committee of Yijishan Hospital and adhered to the ethical standards outlined in the Declaration of Helsinki. Demographic and relevant clinical data, including glycemic, renal, and lipid parameters, were collected from all participants, including glycemic, renal, and lipid parameters.

### 2.2 Inclusion and exclusion criteria

#### 2.2.1 Inclusion criteria

Participants were required to meet the diagnostic criteria for DM as defined by the American Diabetes Association (2021) (17) and for DKD as established by Kidney Disease: Improving Global Outcomes (2020) (18). In addition, supplementary clinical information—such as normal routine urine test results, persistently stable serum CR levels, and no documented history of nephropathy—was reviewed to further exclude individuals with potential overt renal injury (19, 20).

#### 2.2.2 Exclusion criteria

Patients were excluded if they met any of the following criteria: active cardiovascular diseases (e.g., recent acute coronary syndrome, decompensated heart failure, or stroke); a history of malignancy or ongoing anti-cancer therapy; a diagnosis of systemic autoimmune disease; current acute infection; known non-DKD; severe hepatic dysfunction, defined as serum AST or ALT levels exceeding twice the upper limit of normal (2× ULN); secondary hypertension; severe hypertension (systolic blood pressure [SBP] ≥180 mmHg or diastolic blood pressure ≥105 mmHg); or active gastrointestinal bleeding. Additional exclusion criteria included known hypersensitivity to the investigational drug, participation in any other interventional clinical trial within the previous three months, pregnancy or lactation, and any condition that would impair compliance with study procedures.

### 2.3 Grouping criteria

Participants were grouped based on the presence or absence of DKD, in accordance with the KDIGO 2020 diagnostic guidelines. Specifically, individuals were assigned to the DKD group if they exhibited persistent albuminuria (UACR ≥30 mg/g) and/or reduced eGFR <60 mL/min/1.73 m² for more than three months. Those with diabetes but without evidence of renal impairment were assigned to the DM group.

### 2.4 Sample collection and detection

All morning urine samples (collected 7:00–9:00 a.m., after overnight fasting) were aliquoted, frozen at –80 °C, and transported on dry ice to the central laboratory within 24 hours. This standardized protocol ensured consistency across groups and minimized timing-related bias.

U-TXM levels were measured using a fully automated homogeneous enzyme immunoassay (11-Dehydrothromboxane B2 Test Kit; Changsha Boyuan Medical Technology Co., Ltd., China) on a Hitachi 7180 analyzer. The essay employs a liquid-phase competitive immunoassay, where free 11-dehydrothromboxane B2 competes with the enzyme-labeled conjugate for antibody binding. The enzymatic reaction converts NAD^+^ to NADH, with absorbance measured at 340 nm proportional to the U-TXM concentration. Results were normalized to urinary CR and expressed as pg/mg Cr. Assay performance included analytical sensitivity of 1.0 ng/mL, linearity range 0.30–8.00 ng/mL (*r*≥0.990), intra-assay coefficient of variation (CV) ≤10.0%, inter-assay CV ≤15.0%, and accuracy within ±15.0%, according to the manufacturer’s specifications.

Urinary albumin and CR levels were determined via immunoturbidimetry on a BA400 urine analyzer, and UACR was subsequently calculated. Fasting plasma glucose (FPG) was assessed using a glucose oxidase-based assay, and glycated hemoglobin (HbA1c) was quantified by liquid chromatography.

### 2.5 Statistical analysis

Continuous variables with skewed data distributions (such as UACR, U-TXM, blood urea nitrogen [BUN], serum Cr, and triglycerides [TG]) underwent logarithmic transformation. Spearman’s correlation analyses were used to assess associations between U-TXM and renal function markers. Univariate logistic regression was first performed to screen potential predictors of DKD, and variables with *P*<0.1 or clinical relevance were included in the multivariate model. Continuous variables were standardized using Z-scores, and odds ratios (ORs) with 95% confidence intervals (CIs) were calculated. Model calibration was evaluated by the Hosmer–Lemeshow test. Receiver operating characteristic (ROC) curves were generated to assess the diagnostic performance of U-TXM and predictive models. The dataset was randomly split into training (70%) and validation (30%) sets for internal validation. An area under the curve (AUC) >0.7 indicated acceptable discrimination, with the optimal cut-off value determined by the Youden Index.

Group differences in continuous variables were compared using the Mann–Whitney U test or *t*-test, and categorical variables via Chi-square or Fisher’s exact test. Analyses were conducted using SPSS version 26.0 (IBM Corp., NY, USA) and GraphPad Prism version 9.0 (GraphPad Software, CA, USA). A two-sided *P*<0.05 was considered statistically significant. As the analyses were hypothesis-driven, no multiple-comparison adjustments were applied.

## 3 Results

### 3.1 General and clinical characteristics of the study population

A total of 690 participants were enrolled, including 422 in the DM group and 268 in the DKD group. Baseline demographic and clinical characteristics are summarized in **Tables 1**, **2**. The proportion of males was similar between groups (57.6% in both). Disease duration was shorter in the DM group, with 53.4% of patients in the DM group having ≤5 years compared to 38.9% of patients in the DKD. Conversely, longer durations were more common in the DKD group (6 to 10 years, 32.5% in the DKD group compared to 25.9% in the DM group; >10 years, 28.6% in the DKD group compared to 20.6% in the DM group). Body mass index (BMI) distributions differed: 39.9% of patients in the DKD group and 56.3% in the DM group had a BMI in the range of 18.5 to 23.9; 39.2% in the DKD group and 31.0% in the DM group had a BMI in the range of 24 to 27.9; 17.1% of patients in the DKD group and 8.3% in the DM group had a BMI of 28 or higher.

**Table 1:** Demographic characteristics of patients.

Demographic Characteristics		DM	DKD	z/χ²	P-value
Age					
	<40	53 (13.4%)	36 (14.1%)	1.26	0.532
	≥40, <60	197 (49.7%)	116 (45.3%)		
	≥60	146 (36.9%)	104 (40.6%)		
Sex					
	Male	227 (57.6%)	147 (57.6%)	0	0.993
	Female	167 (42.4%)	108 (42.4%)		
DD					
	≤5	171 (53.4%)	79 (38.9%)	10.67	0.005*
	6-10	83 (25.9%)	66 (32.5%)		
	>10	66 (20.6%)	58 (28.6%)		
BMI					
	BMI<18.5	10 (4.4%)	6 (3.8%)	13.1	0.004*
	18.5≤BMI<23.9	129 (56.3%)	63 (39.9%)		
	24≤BMI<27.9	71 (31%)	62 (39.2%)		
	BMI≥28	19 (8.3%)	27 (17.1%)		
BP					
	SBP	129 (119,141.5)	141.2 ± 18.7	-5.81	0*
	DBP	79.2 ± 11.4	81.5 (74,90.3)	-2.61	0.009*
Medical history					
	Hypertension	59 (14%)	106 (39.8%)	59.	0*
	Smoking	67 (20.1%)	25 (11.6%)	6.75	0.009*
	CVD	45 (13.6%)	21 (9.7%)	1.85	0.174
	HBH	2 (0.8%)	1 (0.5%)	0.18	0.670
	HO	12 (4.9%)	10 (4.9%)	0	0.975
Medications					
	Aspirin	16 (3.8%)	26 (9.8%)	10.19	0.001*
	Clopidogrel	14 (3.3%)	8 (3%)	0.05	0.822
	Dual antiplatelet therapy	27 (6.4%)	32 (12.0%)	6.60	0.01*
	Atorvastatin	58 (13.7%)	58 (21.8%)	7.56	0.006*
	Insulin	123 (29.1%)	107 (40.2%)	9	0.003*
	Dapagliflozin	141 (33.4%)	142 (53.4%)	26.88	0*
	Metformin	217 (51.4%)	132 (49.6%)	0.21	0.646
	ACEI/ARB	51 (12.1%)	98 (36.8%)	58.94	0*

1. * indicates *P*<0.05, representing a statistically significant difference. 2. Data are presented as the mean ± SD for normally distributed variables, or as median (IQR) for non-normally distributed variables. 3. The grouping of disease duration and BMI is based on standard definitions. 4. Percentages are calculated based on the total number of people in each group. 5. BMI, body mass index; DKD, diabetic kidney disease; DM, diabetes mellitus; HO, history of tumor; HBH, history of bleeding; IQR, interquartile range; SBP, systolic blood pressure; SD, standard deviation.

**Table 2:** Clinical characteristics of patients in different groups.

Clinical Characteristics		DM	DKD	z/t	P-value
Glucose metabolism indicators					
	HbA1c	7.20 (6.40,9.00)	7.50 (6.40, 9.10)	-1.35	0.176
	FPG	7.25 (6.38, 8.89)	7.47 (6.16, 9.78)	-0.99	0.322
Renal function markers					
	BUN	5.80 (4.69, 6.91)	6.02 (4.92, 7.80)	-2.34	0.02*
	Cr	71.35 (60.18, 81.95)	80.30 (63.88.,99.92)	-4.66	0*
	SUA	294.40 (235,368.23)	302.20 (263.90,398.30)	-3.12	0.002*
	CysC	0.78 (0.66,0.94)	0.93 (0.76,1.29)	-4.54	0*
Liver function markers					
	ALB	44.85 (42.30, 46.83)	44 (40.58, 46.33)	-3.25	0.001*
	Direct Bilirubin	3.90 (2.97, 4.90)	3.39 (2.52, 4.41)	-4.02	0*
	ALT	20(14,28)	19(14,32)	-0.38	0.703
	AST	20(17,26)	20(16,28)	-0.14	0.886
Blood lipids					
	TC	4.51 ± 0.98	4.60 (3.89,5.56)	-2.06	0.039*
	TG	1.30 (0.92,2.01)	1.77 (1.08,2.80)	-4.51	0*
	HDL	1.22 (1.01,1.42)	1.22 (1.04,1.40)	-0.41	0.683
	LDL	2.56± 0.75	2.72± 0.96	-2.11	0.036*
Full blood count					
	WBC	5.95 (5,7.30)	6.8 (5.8, 8.4)	-4.84	0*
	RBC	4.47 ± 0.58	4.58 (3.99, 4.93)	-1.19	0.234
	HB	134.60 ± 18.06	133.53± 22.14	0.55	0.584
	Platelet count	175 (140, 216.25)	198.76 ± 64.70	-3.17	0.002*

*indicates *P*<0.05, indicating a statistically significant difference. Data are presented as the mean ± SD for normally distributed variables, or as median (IQR) for non-normally distributed variables. Alb, albumin; ALT, alanine aminotransferase; AST, aspartate transferase; BUN, blood urea nitrogen; Cr, creatinine; CysC, cystatin C; DBIL, direct bilirubin; DKD, diabetic kidney disease; DM, diabetes mellitus; FPG, fasting plasma glucose; HB, hemoglobin; HbA1, glycated hemoglobin; HDL, high-density lipoprotein; IQR, interquartile range; LDL, low-density lipoprotein; PLT, platelet count; RBC, red blood count; SD, standard deviation; SUA, serum uric acid; TC, total cholesterol; TG, triglyceride; WBC, white blood count.

Medication use was higher in the DKD group for most therapies: aspirin (9.8% vs. 3.8%), dual antiplatelet therapy (12.0% vs. 6.4%), statins (21.8% vs. 13.7%), sodium-glucose cotransporter-2 (SGLT2) inhibitors (53.4% vs. 33.4%), insulin (40.2% vs. 29.1%), and angiotensin-converting enzyme inhibitors/angiotensin receptor blockers (ACEI/ARB; 36.8% vs. 12.1%). Hypertension history was more frequent in DKD (39.8% vs. 14.0%), whereas smoking history was more common in DM (20.1% vs. 11.6%) (**Table 1**).

Clinical and laboratory data showed modest differences. Glycemic control was comparable: HbA1c levels in the DKD group were 7.50 (6.40, 9.10), compared to 7.20 (6.40, 9.00) in the DM group; FPG levels in the DKD group were 7.47 (6.16, 9.78), compared with 7.25 (6.38, 8.89) in the DM group. Renal markers were elevated in DKD: BUN levels in the DKD group were 6.02 (4.92, 7.80) compared to 5.80 (4.69, 6.91) in the DM group; serum Cr levels in the DKD and DM groups were 80.30 (63.88, 99.92) and 71.35 (60.18, 81.95), respectively. Platelet counts were higher in the DKD group (200×10^9^[154,232.5]) compared to the DM group (175.00 ×10^9^ [140.00, 216.25]) (**Table 2**).

### 3.2 Elevated U-TXM levels in patients with DKD

U-TXM levels were significantly higher in the DKD group than in the DM group, as confirmed by stratified analysis. By disease duration (≥5 years), U-TXM levels were significantly higher in the DKD group than in the DM group (Z=-4.92, *P*<0.001). Similar patterns occurred for BMI (18.5–23.9 kg/m²: Z=-4.16, *P*<0.001), smoking history (Z=-2.68, *P*=0.007), and atorvastatin use (Z=-2.26, *P*=0.023).

Glucose metabolism indices indicated that U-TXM levels were significantly higher in DKD patients in both the FPG ≤6.1mmol/L (Z=-2.74, *P*=0.006) and FPG >6.1 mmol/L (Z=-4.75, *P*<0.001) subgroups. U-TXM levels were also elevated in DKD patients irrespective of HbA1c levels (HbA1c ≥7%: Z=-3.71, *P*<0.001; HbA1c <7%: Z=-4.01, *P*<0.001). Renal function stratification also revealed significantly higher U-TXM levels in DKD patients regardless of serum Cr (>70 μmol/L: Z=-4.39, *P*<0.001; <70 μmol/L: Z=-4.96, *P*<0.001. Lipid profile stratification further showed that U-TXM levels were significantly higher in the DKD group: low-density lipoprotein cholesterol (LDL-C) <1.4 mmol/L (Z=-1.95, *P*=0.05), microalbumin (mAlb) 40–55 g/L (*P*<0.001), and platelet count 100–300 × 10^9^/L (*P*<0.001) (**Table 3**).

**Table 3:** Comparison of U-TXM concentrations between DM and DKD patients.

U-TXM Levels in DM vs DKD			DM	DKD	Z	P-value
Overview			960.44 (680.78,1291.14)	1158.05 (823.44,1697.38)	-5.472	0*
General characteristics						
	DD	≤5 years	911.45 (680.27, 1223.53)	1260.73 (913.91, 1883.94)	-4.922	0*
		6–10 years	989.36 (689.11, 1435.25)	1113.27 (846.80, 1722.82)	-1.762	0.078
		>10 years	1025.72 ± 448.08	1168.51 (752.66, 1660.23)	-1.988	0.047*
	BMI	<18.5	1251.87 (814.77, 1357.91)	1625.34 ± 940.84	-1.193	0.233
		18.5-23.9	1000.61 (686.05, 1310.97)	1382.66 (906.52, 2154.78)	-4.165	0*
		24-27.9	979.40 (770.12, 1292.81)	1100.89 (873.96, 1583.99)	-1.646	0.1
		≥28	941.51 (680.96, 1294.41)	1231.76 ± 587.70	-1.037	0.3
	Hypertension		1046.44 (704.07, 1476.14)	1063.37 (741.99, 1684.57)	-0.738	0.461
	Smoking		1014.62 ± 359.02	1461.63 ± 792.85	-2.681	0.007*
Medications						
	Aspirin		666.93 (491.38, 1325.51)	963.00 (700.11, 1258.60)	-1.502	0.133
	Atorvastatin		1029.24 ± 571.05	1106.30 (859.24, 1710.31)	-2.269	0.023*
	Insulin		1050.94 (766.99, 1379.75)	1100.977 (856.74, 1673.11)	-1.537	0.124
	ACEI/ARB		983.43 (704.07, 1423.55)	1093.86 (741.99, 1684.57)	-0.948	0.343
Glucose metabolism indicators						
	FPG	≤6.1	898.51 (642.02, 1178.46)	1127.86 (743.04, 1682.24)	-2.745	0.006*
		>6.1	981.15 (696.12, 1297.07)	1176.56 (859.54, 1729.58)	-4.756	0*
	HbA1c	<7	837.05 (619.26, 1132.39)	1061.89 (761.48, 1540.21)	-4.015	0*
		≥7	1040.22 (789.68, 1372.13)	1209.98 (880.78, 1730.09)	-3.715	0*
Renal function markers						
	UA	≤420	975.40 (702.59, 1277.86)	1124.05 (827.87, 1727.60)	-4.324	0*
		>420	771.99 (606.84, 1076.78)	1214.40 (884.32, 1590.94)	-4.019	0*
	CysC	<0.57	889.23 ± 454.87	1472.13 ± 779.43	-2.238	0.025*
		0.57-0.97	920.95 (721.23, 1299.74)	1205.22 (836.86, 1679.96)	-3.234	0.001*
		>0.97	989.36 (763.45, 1252.86)	1127.86 (851.38, 1862.69)	-1.477	0.14
	BUN	≤7.5	968.04 (722.110, 1275.08)	1211.13 (899.13, 1756.77)	-5.78	0*
		>7.5	900.12 (620.26, 1292.97)	954.37 (703.87, 1313.32)	-1.356	0.175
	Cr	≤73	1043.34 (818.93, 1372.81)	1332.55 (955.69, 1827.41)	-4.391	0*
		>73	826.78 (612.12, 1156.24)	1046.29 (740.93, 1491.44)	-4.116	0*
Blood lipids						
	TC	≤5.7	949.59 (680.78, 1277.42)	1133.70 (840.56, 1692.09)	-4.967	0*
		>5.7	1075.48 ± 386.59	1274.03 ± 642.08	-1.236	0.216
	LDL-C	<1.4	815.41 (607.84, 1139.67)	1298.57 ± 616.13	-1.958	0.05*
		1.4-3.1	949.38 (676.43, 1280.28)	1133.70 (877.95, 1731.10)	-4.601	0*
		>3.1	1009.65 (767.78, 1343.12)	1270.91 ± 651.61	-1.742	0.081
	TG	≤2.3	964.25 (678.69, 1279.51)	1177.76 (879.05, 1840.76)	-5.078	0*
		>2.3	966.75 (749.03, 1299.53)	1110.56 (803.97, 1507.49)	-1.427	0.153
Liver function markers						
	Alb	<40	965.42 (705.00, 1305.23)	1114.69 (879.36, 1963.34)	-1.921	0.055
		40-55	954.70 (693.03, 1278.30)	1177.76 (815.03, 1667.71)	-4.67	0*
		>55	1057.97 ± 524.18	1494.63 ± 906.95	-1.223	0.221
Full blood count						
	Platelet count	<100	1178.79 ± 376.85	1094.85 ± 402.30	-0.563	0.573
		100-300	967.12 (722.97, 1291.70)	1191.78 (873.41, 1727.60)	-4.887	0*
		>300	1108.12 ± 534.49	1802.34 ± 872.20	-2.135	0.033*

Data are presented as mean ± SD for normally distributed variables, or as median (IQR) for non-normally distributed variables, and differences between groups were tested using the Mann-Whitney U test, with corresponding z-values and P-values reported. * denotes *P*<0.05. ACEI/ARB, angiotensin-converting enzyme inhibitors/angiotensin receptor blockers; Alb, albumin; BUN, blood urea nitrogen; Cr, creatinine; CysC, cystatin C; DD, duration of diabetes; DKD, diabetic kidney disease; DM, diabetes mellitus; FPG, fasting plasma glucose; HB, hemoglobin; HbA1, glycated hemoglobin; IQR, interquartile range; LDL-C, low-density lipoprotein cholesterol; PLT, platelet count; SD, standard deviation; SUA, serum uric acid; TC, total cholesterol; TG, triglyceride; UA, urinalysis.

### 3.3 Correlation analysis of U-TXM with UACR and renal function markers

**Figure 1:**
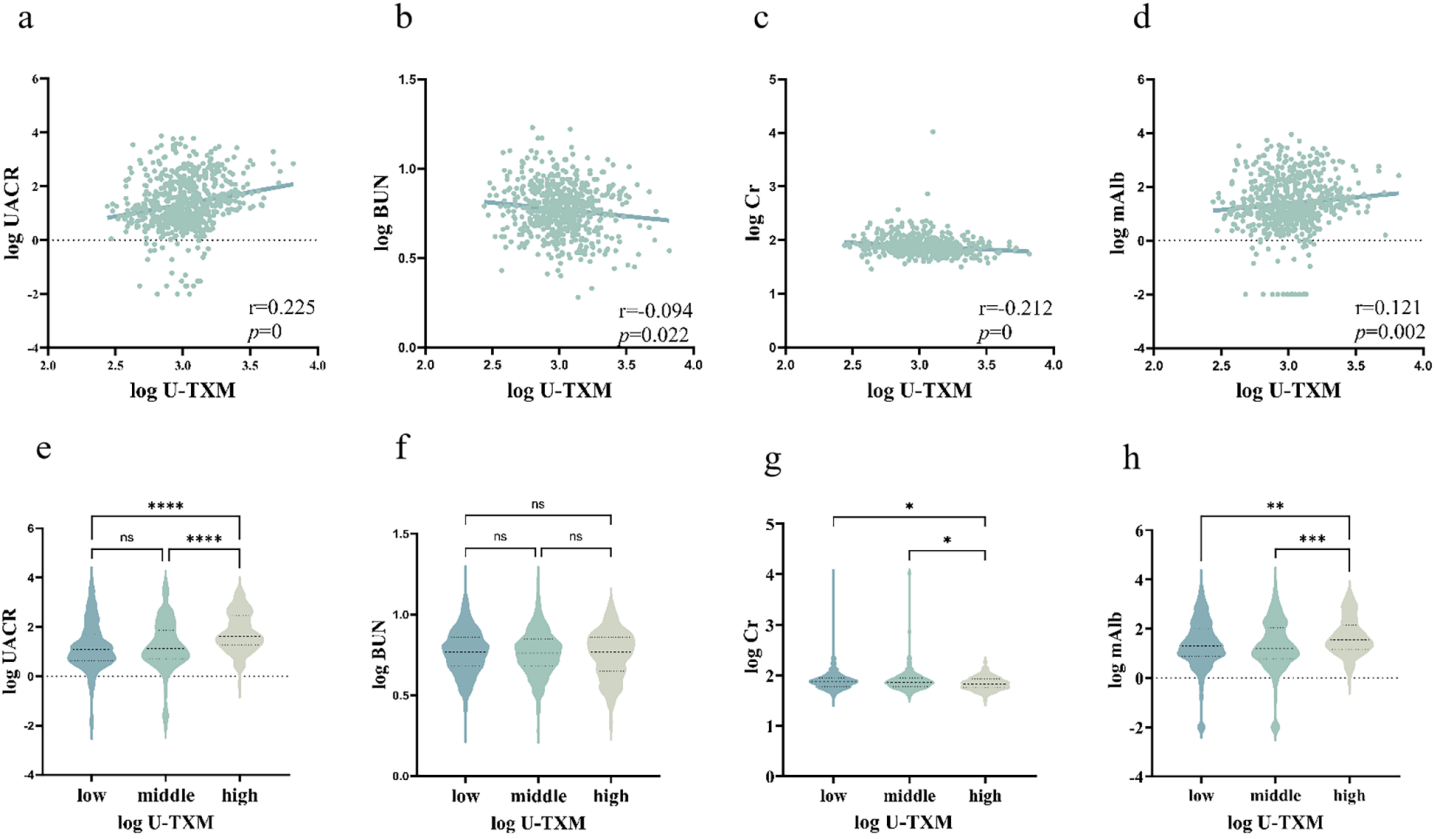
Correlations between U-TXM and renal function markers.**(a)** Scatter plot of log(U-TXM) vs. log(UACR); **(b)** scatter plot of log(U-TXM) vs. log(BUN); **(c)** scatter plot of log(U-TXM) vs. log(serum creatinine, Cr); **(d)** scatter plot of log(U-TXM) vs. log(microalbumin, mAlb); **(e)** violin plot of UACR across low/middle/high U-TXM groups; **(f)** violin plot of BUN across low/middle/high U-TXM groups; **(g)** violin plot of Cr across low/middle/high U-TXM groups; **(h)** violin plot of mAlb across low/middle/high U-TXM groups.Panels **(a–d)** display Spearman correlation coefficients (r) with P values; panels **(e–h)** show distributions stratified by U-TXM. Variables were log-transformed where applicable. Differences in the plots are indicated by asterisks: ns denotes P>0.05, * denotes P<0.05, ** denotes P<0.01, *** denotes P<0.001, **** denotes P<0.0001.

Intergroup and correlation analyses revealed a significant positive correlation between U-TXM and UACR (**Figures 1a, e**), suggesting that as U-TXM levels increase, the UACR also rises. Notable differences in the UACR were observed among different U-TXM concentration subgroups, with the high-U-TXM concentration group exhibiting a more pronounced elevation in the UACR compared to the low concentration group. This finding further validates the strong association between U-TXM and UACR. A weak correlation was found between U-TXM and BUN (**Figures 1b, f**). The levels of BUN differed between the low and high-U-TXM concentration groups. The elevation in BUN was more pronounced in the low-U-TXM concentration group. Similarly, analysis of the correlation between U-TXM and Cr demonstrated a weak relationship (**Figures 1c, g**). Differences in Cr levels were observed between U-TXM subgroups, with the low-U-TXM concentration group exhibiting a more pronounced elevation in Cr levels. Further analysis of the relationship between U-TXM and mAlb showed a weak positive correlation between these two markers (**Figures 1d, h**). Differences in mAlb levels were also observed in patients with different U-TXM concentrations, with higher mAlb levels detected in the high-U-TXM concentration subgroup. This result was consistent with the correlation analysis based on scatter plots. Overall, U-TXM is correlated with renal function markers such as UACR and mAlb. While the correlation between U-TXM and BUN or Cr was weak, intergroup differences were observed.

### 3.4 U-TXM is an effective predictor for DKD

**Figure 2:**
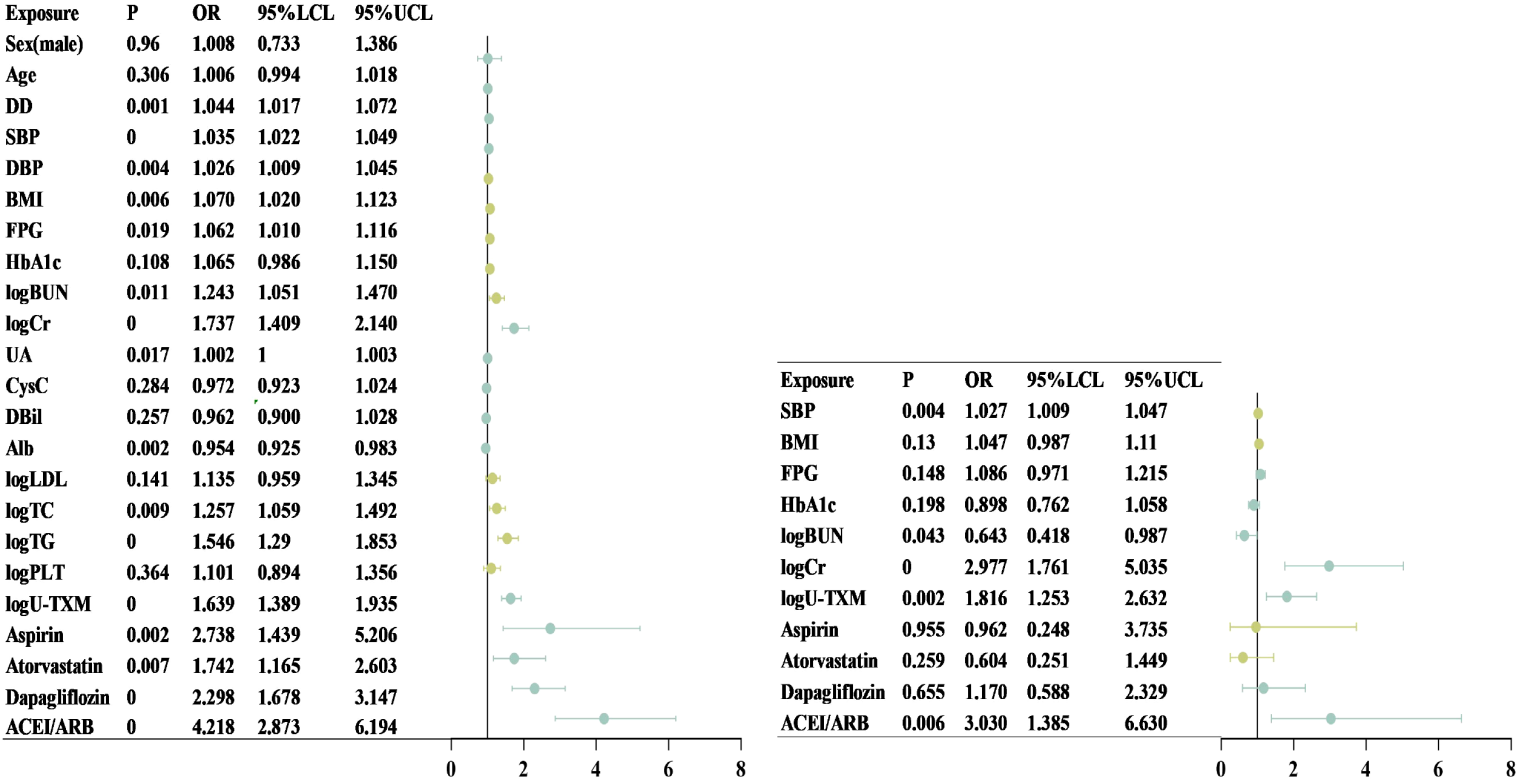
Forest plot of univariate and multivariate logistic regression analysis. This chart shows the effect of different clinical variables on the target outcome in univariate (left) and multifactorial (right) Logistic regression analyses. The dot represents the OR and the horizontal line represents the 95% CI. Chart on the left (single factor analysis): Including Sex, Age, DD, SBP, DBP, BMI, FPG, HbA1c, logBUN, logCr, UA, CysC, FPG, DBil, Alb, logLDL, logTC, logTG, logPLT, and LOU-TXM. Among them, the variables with a *P*-value <0.05 were statistically significant. Figure on the right (multivariate analysis): After adjusting for other variables, SBP, logBUN, logCr, and logU-TXM were still significantly associated with the target outcome (*P*<0.05), while the effects of BMI, FPG, and HbA1c were no longer significant after adjustment. Alb, albumin; BMI, body mass index; BUN, blood urea nitrogen; CI, confidence interval; Cr, creatinine; CysC, cystatin C; DBP, diastolic blood pressure; DBil, direct bilirubin; DD, disease duration; FPG, fasting plasma glucose; HbA1c, glycated hemoglobin; LDL, low-density lipoprotein cholesterol; logBUN, log-transformed blood urea nitrogen; logCr, log-transformed creatinine; logLDL, log-transformed low-density lipoprotein cholesterol; logPLT, log-transformed platelet count; logTC, log-transformed total cholesterol; logTG, log-transformed triglycerides; logU-TXM, log-transformed urinary 11-dehydrothromboxane B; OR, odds ratio; SBP, systolic blood pressure; TC, total cholesterol; TG, triglycerides; UA, uric acid; U-TXM, urinary 11-dehydrothromboxane B.

After logarithmic transformation and standardization of the data, univariate logistic regression analysis demonstrated that U-TXM (OR=1.639, 95% CI: 1.389–1.935, *P*<0.001) was significantly associated with an increased risk of DKD (**Figure 2**, left). Other significant predictors included serum Cr (OR=1.737, 95% CI: 1.409–2.140, *P*<0.001), TG (OR=1.546, 95% CI: 1.290–1.853, *P*<0.001), TC (OR=1.257, 95% CI: 1.059–1.492, *P*=0.009), BUN (OR=1.243, 95% CI: 1.051–1.470, *P*=0.011), and UA (OR=1.002, 95% CI: 1.000–1.003, *P*=0.017). In addition, the use of aspirin (OR=2.738, 95% CI: 1.439–5.206, *P*=0.002), atorvastatin (OR=1.742, 95% CI: 1.165–2.603, *P*=0.007), dapagliflozin (OR=2.298, 95% CI: 1.678–3.147, *P*<0.001), and ACEI/ARB (OR=4.218, 95% CI: 2.873–6.194, *P*<0.001) were significantly associated with DKD. These findings highlighted the diagnostic potential of renal function markers, lipid metabolism indicators, and medications commonly used in relation to DKD risk. In contrast, sex, age, HbA1c, CysC, and direct bilirubin did not reach statistical significance in univariate analysis.

To further examine independent associations, multivariate regression analysis was performed (**Figure 2**, right). After adjusting for potential confounders, SBP (OR=1.027, 95% CI: 1.009–1.047, *P*=0.004) remained significantly associated with DKD, indicating that each 1 mmHg increase in SBP raised the risk by 2.7%. Elevated Cr (OR=2.977, 95% CI: 1.761–5.035, *P*<0.001) also emerged as a strong independent risk factor. Interestingly, BUN demonstrated an inverse association (OR=0.643, 95% CI: 0.418–0.987, *P*=0.043), suggesting a potential protective effect after adjusting for other variables. Importantly, U-TXM remained independently associated with DKD (OR=1.816, 95% CI: 1.253–2.632, *P*=0.002), supporting its role as a robust predictive biomarker. In contrast, aspirin, atorvastatin, and dapagliflozin were no longer significant after adjustment, whereas ACEI/ARB use (OR=3.030, 95% CI: 1.385–6.630, *P*=0.006) remained independently associated with DKD occurrence. Although BMI, FPG, and HbA1c lost statistical significance, they were retained in the multivariate model given their clinical relevance in DM and DKD. Collectively, these findings suggest that U-TXM serves as a significant independent risk factor with potential predictive value for early DKD diagnosis, even after accounting for medication use and other covariates.

### 3.5 Diagnostic performance of U-TXM for early detection of DKD

**Figure 3:**
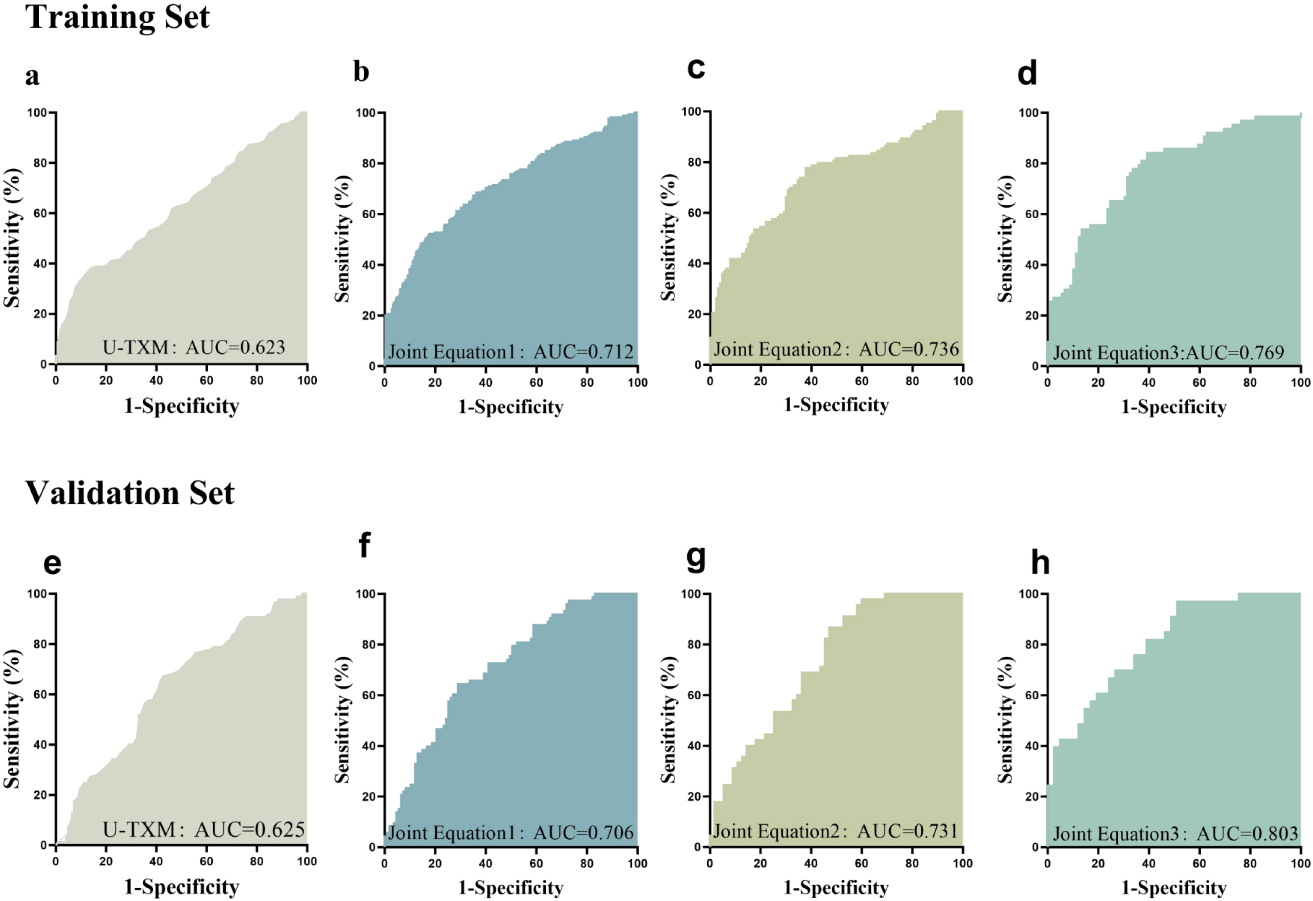
ROC curve analysis. This figure presents the ROC curves evaluating the predictive performance of different variables for the target outcome. The AUC was 0.623 in the training set **(a)** and 0.625 in the validation set **(e)**. SimultaneousEquation 1: it(P) = -0.424 + 0.768 × Cr + 0.685 × U-TXM. This model achieved an AUC of 0.712 in the training set **(b)** and 0.706 in the validation set **(f)**. Simultaneous Equation 2: it(P) = -4.438 + 0.903 × Cr - 0.251 × BUN + 0.563 × U-TXM + 0.031 × SBP. The AUC of this model was 0.736 in the training set **(c)** and 0.731 in the validation set **(g)**. Simultaneous Equation 3: it(P) = -5.733 + 1.051 × Cr - 0.420 × BUN + 0.576 × U-TXM + 0.031 × SBP + 0.054 × BMI - 0.137 × HbA1c + 0.101 × FPG. This model achieved an AUC of 0.769 in the training set **(d)** and 0.803 in the validation set **(h)**. The x-axis represents 1 - Specificity, while the y-axis represents Sensitivity. Different colored curves indicate different models or variables, with a larger AUC signifying stronger predictive ability. The ROC curve is used to assess the classification performance of different biomarkers or models. AUC values closer to 1 indicate higher predictive accuracy. AUC, area under the curve; ROC, receiver operating characteristic.

The AUC was 0.623 in the training set (**Figure 3a**) and 0.625 in the validation set (v **Figure 3b**). Based on ROC analysis, the optimal cut-off value of U-TXM for differentiating DKD from DM was 1430.27 pg/mg Cr, with a sensitivity of 0.377 and specificity of 0.866. The AUC was 0.6248 (95% CI: 0.5721–0.6775, *P*<0.0001), reflecting the limited diagnostic accuracy of U-TXM alone. To enhance diagnostic efficiency, we incorporated multiple factors to establish a predictive model using simultaneous equations. The validity of the model was evaluated through ROC curve analysis. Simultaneous Equation 1: it(P) = -0.424 + 0.768 × Cr + 0.685 × U-TXM. This model achieved an AUC of 0.712 in the training set and 0.706 in the validation set. Although it includes the fewest variables, its predictive performance is relatively weak, making it suitable only when data are limited. Simultaneous Equation 2: it(P) = -4.438 + 0.903 × Cr - 0.251 × BUN+0.563 × U-TXM + 0.031 × SBP. The AUC of this model was 0.736 in the training set and 0.731 in the validation set. While slightly less predictive than Simultaneous Equation 1, it included the additional variables Cr, BUN, U-TXM, and SBP, offering a simplified but more effective model than the single-variable model. Simultaneous Equation 3: it(P) = -5.733 + 1.051 × Cr - 0.420 × BUN+0.576 × U-TXM + 0.031 × SBP+0.054 × BMI - 0.137 × HbA1c + 0.101 × FPG. This model demonstrated the highest predictive performance and good generalization ability, with an AUC of 0.769 in the training set and 0.803 in the validation set. In summary, although U-TXM alone showed limited diagnostic value for DKD (AUC=0.623), its performance improved significantly in a multivariate model incorporating renal, metabolic, and blood pressure indicators. Simultaneous Equation 3 is recommended as the optimal predictive model due to its superior predictive ability and generalizability, while Simultaneous Equation 2 may serve as an alternative when a more simplified model is required. These findings support the potential use of U-TXM-based diagnostic models for early detection of DKD.

**Figure 4:**
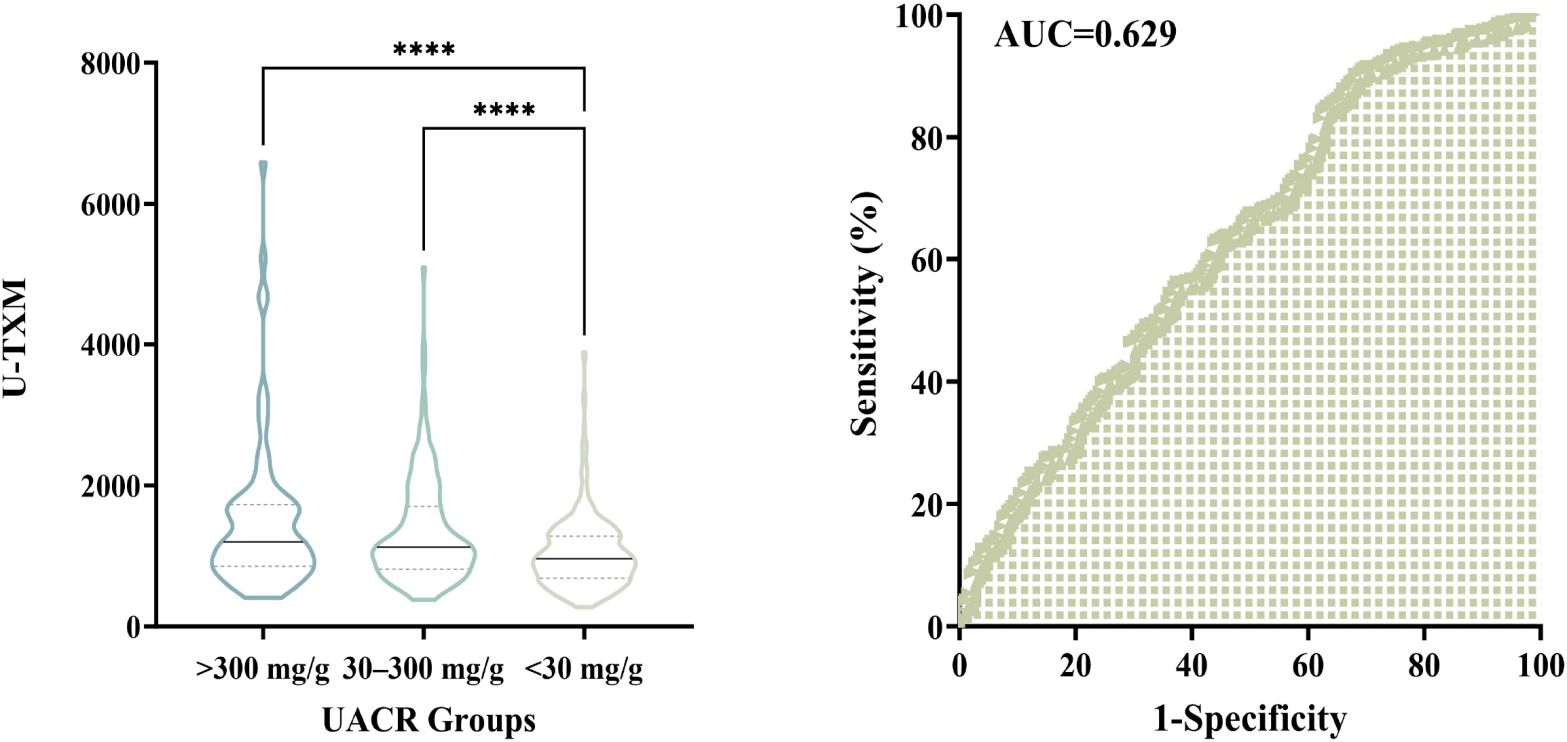
Association between urinary U-TXM levels and UACR groups, and diagnostic utility of U-TXM for DKD progression. Left panel: Violin plot showing the distribution of urinary U-TXM levels across UACR groups: A1 (<30 mg/g), A2 (30–300 mg/g), and A3 (>300 mg/g). U-TXM levels increase progressively from A1 to A3, with statistically significant differences observed between A1 and A2 (P<0.0001), as well as between A1 and A3 (P<0.0001). Right panel: ROC curve for U-TXM in discriminating patients with elevated albuminuria (A2/A3, UACR ≥30 mg/g) from those with normal albumin levels (A1). The AUC is 0.629, indicating statistical significance. These findings highlight the potential of U-TXM as a supplementary biomarker for assessing DKD progression. “****” indicates p < 0.0001, as per our statistical analysis.

To further investigate the association between urinary U-TXM levels and DKD severity, patients were stratified according to UACR into three categories: A1 (<30 mg/g), A2 (30–300 mg/g), and A3 (>300 mg/g). As shown in **Figure 4**, left, U-TXM levels increased progressively across the A1 to A3 groups, with statistically significant differences between A1 and A2 (*P*<0.0001), as well as A1 and A3 (*P*<0.0001). These results indicate a positive correlation between U-TXM excretion and albuminuria severity.

We further performed ROC curve analysis to assess the diagnostic utility of U-TXM in discriminating patients with elevated albuminuria (A2/A3, UACR ≥30 mg/g) from those with normal albumin levels (A1). As depicted in **Figure 4**, right, the AUC was 0.629, indicating statistical significance. These findings support the potential role of U-TXM as a supplementary biomarker for DKD progression.

## 4 Discussion

**Figure 5:**
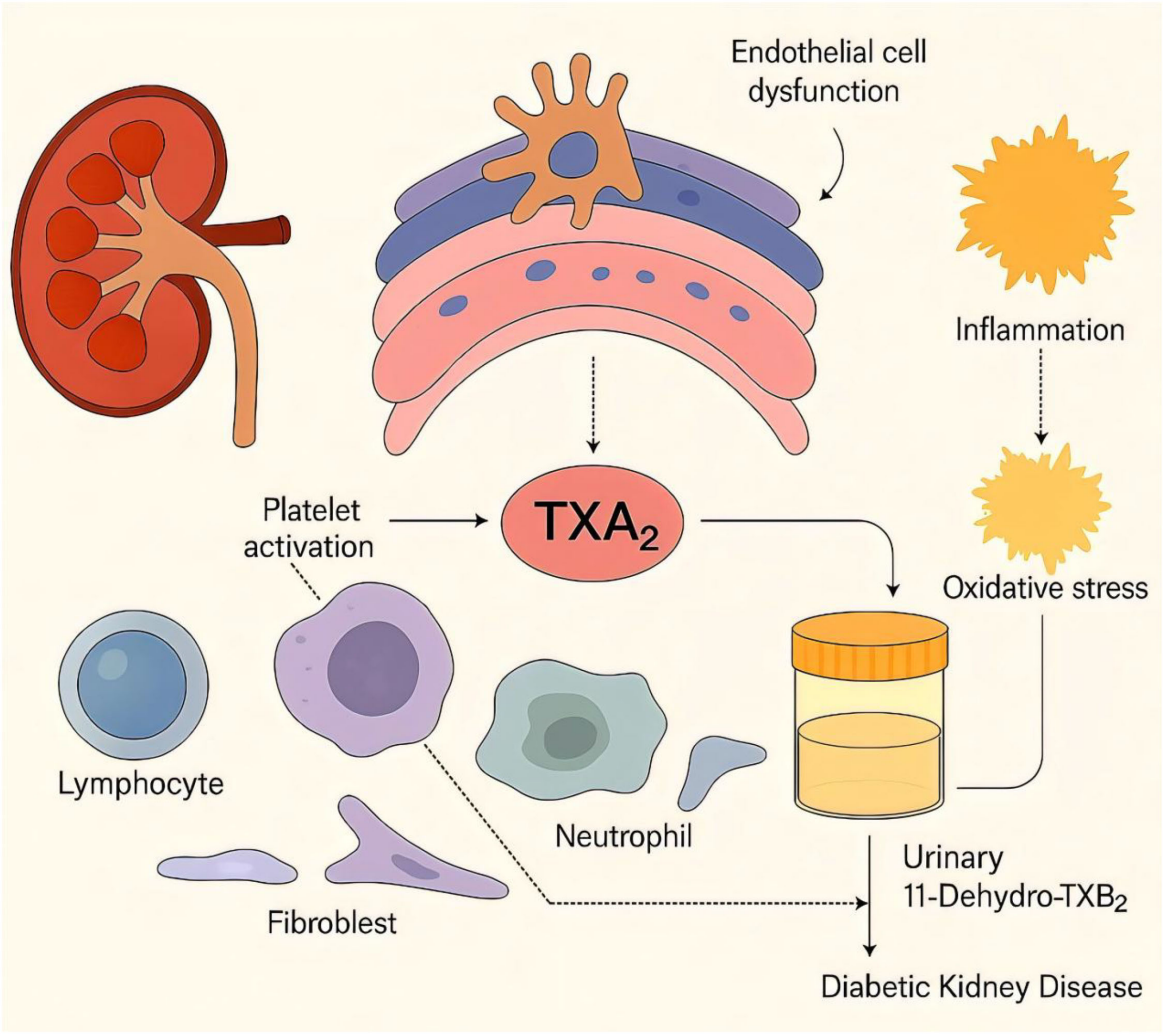
Schematic illustration of the pathways linking urinary 11-dehydrothromboxane B_2_ (U-TXM) to diabetic kidney disease (DKD). Platelet activation increases thromboxane A_2_ (TXA_2_) generation, which promotes renal inflammation and contributes to endothelial/podocyte dysfunction and fibrotic signaling, thereby facilitating DKD progression (6, 7, 12–14). TXA_2_ is rapidly hydrolyzed to TXB_2_ and further metabolized to the stable urinary metabolite U-TXM, which reflects *in-vivo* TXA_2_ biosynthesis and platelet activation (15). Prior studies associate elevated U-TXM with heightened inflammatory/vascular risk and adverse prognosis, supporting its biomarker potential (21, 23, 24). In diabetes, platelet hyperreactivity and treatment factors may modulate TXA_2_/U-TXM levels (25, 26, 28).Solid arrows indicate confirmed mechanisms, while dashed arrows represent hypothetical links.

DKD is one of the most prevalent comorbidities of DM and the leading cause of ESRD. Currently, UACR is the most commonly used clinical diagnostic marker for DKD. However, the early stages of DKD are often asymptomatic, and by the time albuminuria is detected, renal lesions may have already progressed to an advanced stage, leading to a rapid decline in kidney function toward ESRD (1). The progression of DKD may be delayed or even halted by early detection and intervention. However, a major limitation of current diagnostic methods is their inability to detect DKD in non‐albuminuric phenotypes, which are becoming increasingly prevalent and lack targeted therapies. U-TXM is a metabolite of TXA2 (21). Elevated U-TXM levels are strongly associated with inflammatory conditions (e.g., coronary heart diseases, atherosclerosis), vascular inflammation, and poor prognosis (22). In patients with DKD, pathological processes such as chronic inflammation, oxidative stress, and vascular endothelial dysfunction may lead to increased platelet activation and subsequent U-TXM production. Thus, U-TXM may serve as an indicator of systemic inflammation in DKD patients. Additionally, U-TXM has been recognized as a potential biomarker for predicting disease prognosis (23, 24) (**Figure 5**).

Previous studies have identified several factors significantly associated with elevated U-TXM levels, such as advanced age, female sex, history of peripheral artery disease, and use of aspirin. In patients with DM, U-TXM levels are generally higher than in healthy controls (25, 26). However, it remains unclear whether U-TXM levels can further increase after the onset of DKD in DM patients. In this study, we found that U-TXM levels were significantly elevated in DM patients with DKD compared to those without DKD. The increased concentration of U-TXM was closely associated with the pathological features of DKD, suggesting that this biomarker may reflect metabolic alterations and inflammatory characteristics of DKD. In patients with shorter disease duration (≤5 years), U-TXM levels in the DKD group were significantly higher than those in the DM group. This finding suggests that U-TXM may be more sensitive in detecting early renal lesions. These findings support the potential use of U-TXM as a biomarker for the early prediction of DKD risk. In patients with more than 10 years of disease duration, although U-TXM levels remained significantly elevated in the DKD group, the intergroup differences diminished over time. This may be due to the diminishing effectiveness of metabolic compensatory mechanisms in patients with prolonged disease duration. Previous studies have shown that U-TXM levels increase with the duration of DM (27). Furthermore, patients with a longer disease duration tend to be older, and age is also associated with elevated U-TXM levels (25). Therefore, the combined effects of prolonged disease duration and increased age may offset the increase in U-TXM levels due to DKD.

In the stratification of patients with less impaired renal function, U-TXM levels were significantly higher in the DKD group, suggesting that this biomarker may reflect the early stages of renal function impairment. This finding is consistent with the early upward trend observed in CysC and Cr levels, indicating that U-TXM can be a useful monitoring index for patients with minor renal impairment. However, as renal function continued to deteriorate, especially when CysC exceeded 0.97 mg/L, the intergroup differences in U-TXM levels tended to diminish. This may be due to the saturation of inflammatory and oxidative stress responses during severe kidney injury, which reduces the diagnostic sensitivity of U-TXM. Petrucci et al. found that elevated U-TXM levels were associated with type 2 DM, UACR ≥3 mg/mmol, and a higher glomerular filtration rate (25), which is consistent with our findings. In patients with a history of smoking, U-TXM concentrations were significantly higher, suggesting smoking, as an inflammatory inducer, may exacerbate inflammation and oxidative stress levels associated with DKD, thereby promoting U-TXM secretion. This result supports an earlier study that identified smoking as an independent factor associated with increased urinary U-TXM concentrations (21). Stratified lipid analysis showed that U-TXM was significantly elevated in DKD patients with low LDL-C (<1.4 mmol/L) and low TG (<2.3 mmol/L), suggesting that U-TXM can effectively reflect the pathological state of inflammation and metabolic disorders, even in patients with low lipid levels. Statins have been shown to inhibit platelet hyperreactivity and lower U-TXM concentrations in patients with type IIa hypercholesterolemia (26, 28), and improve endothelial function in patients with coronary artery disease (26, 28). By reducing tissue factor expression and thrombin release from dysfunctional endothelium, statin therapy may attenuate platelet activation and aggregation (26, 28). Despite the lipid-lowering and anti-inflammatory effects of statins, U-TXM levels in DKD patients remain significantly higher than in DM patients, indicating inflammation in DKD patients even with statin treatment. Therefore, U-TXM may serve as an indicator of inflammation in the context of statin therapy. The study by Eikelboom et al. also found that hypercholesterolemia and stain treatment were independently associated with increased urinary U-TXM concentrations, which is consistent with our findings (26, 28).

Many variables are independently associated with U-TXM levels (25, 26). However, the relationship between UACR and U-TXM remains unclear. In this study, we found a significantly positive correlation between U-TXM and UACR, indicating that U-TXM could be a key marker of glomerular injury, particularly in patients with abnormal urinary protein excretion. The weak relationship between U-TXM and renal function markers (e.g., BUN and Cr) suggests that U-TXM may have limited independence in assessing overall renal function. However, some differences were observed in specific concentration groups. COX1 and COX2 are also associated with the concentrations of U-TXM (26, 28). Inhibition of COX-2 has been found to reduce the endothelial synthesis of prostacyclin, which can adversely affect renal function. This may explain the observed differences between U-TXM and traditional renal function markers. Multivariate regression analysis revealed that the OR for U-TXM in relation to UACR was 1.778, suggesting that U-TXM is an independent risk factor for DKD. Further ROC curve analysis confirmed the predictive value of U-TXM for DKD. Although U-TXM showed statistical significance in distinguishing DKD from DM, its standalone diagnostic value was modest (AUC=0.623), suggesting limited utility as an independent marker. However, when combined with renal function, metabolic, and blood pressure indicators in a multivariate model, diagnostic performance improved markedly (AUC=0.803). These findings highlight the potential of U-TXM as part of a multi-marker panel for early DKD detection and risk assessment. In this study, Simultaneous Equation 3 is recommended as the optimal model for risk assessment and clinical prediction of DKD because of its strong predictive power and robust generalization performance.

Despite the promising findings, several limitations should be acknowledged. First, the cross-sectional and single-center design restricts the ability to infer causality or temporal dynamics. Although U-TXM levels were associated with DKD severity, prospective longitudinal studies are needed to evaluate whether elevated U-TXM predicts renal progression. Ideally, such cohorts should include patients with baseline normoalbuminuria (UACR<30 mg/g) and preserved renal function (eGFR≥60 mL/min/1.73 m²), followed over 3–5 years to assess whether U-TXM levels correlate with future eGFR decline or onset of proteinuria. Second, the generalizability of our results is limited due to the single-center population. Multicenter validation in diverse cohorts is needed to confirm our findings across different ethnic, clinical, and healthcare backgrounds. Third, analytical limitations of U-TXM measurement should be considered. Although ELISA assays are widely available and cost-effective compared to omics platforms, they pose potential issues with cross-reactivity and measurement variability, especially in routine clinical use. Fourth, significant differences in medication use (e.g., aspirin, statins, SGLT2 inhibitors, ACEI/ARB; all *P*<0.01) between groups may have influenced U-TXM levels via effects on platelet activity, inflammation, or renal function. Although we adjusted for these confounders in multivariate analyses, residual confounding cannot be ruled out. Fifth, comorbidities such as hypertension complicate the interpretation of U-TXM changes, particularly in distinguishing DKD from hypertensive nephrosclerosis. Renal biopsy remains the diagnostic gold standard, but was not performed in this study. Lastly, although U-TXM exhibited a certain degree of diagnostic performance in the overall DKD population for albuminuria-based DKD classification (AUC=0.63), its independent value in non-albuminuric DKD remains unclear. In our study, a subset of patients (n=24) presented with reduced eGFR (<60 mL/min/1.73 m²) but normal UACR levels, suggestive of early renal impairment without albuminuria. Due to the small sample size, subgroup analyses were not feasible. Future studies should specifically recruit and analyze non-albuminuric DKD populations to determine whether U-TXM can aid in early detection.

In conclusion, this study is the first to systematically investigate the potential value of U-TXM in DKD, especially its application in early diagnosis. The results show high sensitivity in patients with a short disease duration and good metabolic status, suggesting that U-TXM could be used for screening and dynamic monitoring of high-risk individuals. In addition, the role of U-TXM in smokers and patients receiving statin therapy further highlights its importance in assessing inflammatory status.

## Data Availability

The raw data supporting the conclusions of this article will be made available by the authors, without undue reservation.
